# Bailout subclavian artery stenting in a sick child with iatrogenic subclavian artery perforation: a case report

**DOI:** 10.1186/s43044-020-00056-2

**Published:** 2020-04-07

**Authors:** Sanjeev Naganur, Krishna Santosh, Bhupendra Kumar Sihag, Atit A Gawalkar, Krishna Prasad

**Affiliations:** Department of Cardiology, Post Graduate Institute of Medical Education and Research, Chandigarh, India

**Keywords:** Iatrogenic subclavian artery injury, Internal jugular vein catheterization, Endovascular management, Covered stent, Case report

## Abstract

**Background:**

Subclavian artery injury during internal jugular vein catheterization is a rare yet potentially life-threatening complication leading to hemothorax and exsanguination. The percutaneous endovascular approach offers a less invasive and effective alternative to the high-risk surgical repair in emergent situations.

**Case presentation:**

We present a case of a 6-year-old child suffering from hemolytic uremic syndrome requiring urgent hemodialysis, for which IJV (internal jugular vein) cannulation was attempted. This procedure led to iatrogenic subclavian arterial perforation causing massive hemothorax with hemodynamic compromise. CT angiogram showed a through and through perforation in the first part of right subclavian artery between common carotid and vertebral artery. A definitive assessment of the extent of ongoing leak was made through an invasive angiogram in the catheterization laboratory. The perforation was successfully closed percutaneously with a covered stent without compromising any branch vessels.

**Conclusion:**

Arterial injury although rare is a potentially life-threatening complication of IJV cannulation which warrants immediate attention and corrective measures. Ultrasound guidance can reduce the risk of such life-threatening complications. Percutaneous management offers a less invasive, less time consuming, and effective alternative in critically ill patients in emergency situations.

## Background

Central line placement has become the standard of care in critical care settings. Complications during internal jugular vein (IJV) cannulation occur in 15% of patients [1]. Arterial injury can cause significant hemodynamic compromise, when not recognized in time. Open surgical repair requires extensive dissection in a critically ill patient, precluding its universal use. Therefore, endovascular intervention offers a less invasive and effective alternative to open surgical repair in emergencies.

## Case presentation

A 6-year-old child, weighing 15 kg, was diagnosed to have hemolytic uremic syndrome (HUS) and was advised for hemodialysis. Right IJV was selected for dialysis catheter insertion. After insertion, no blood was aspirable from the catheter raising doubt about the position of catheter tip. Therefore, the catheter was removed after which there was a sudden hemodynamic deterioration. Massive hemothorax was suspected which was confirmed by chest radiograph. After resuscitation with multiple transfusions (6 units of packed red cells) and intercoastal drain insertion, CT was performed. It showed leak from a through and through rent in the first part of right subclavian artery (SCA) between common carotid artery (CCA) and vertebral artery (VA). The emergency multidisciplinary team consisting of nephrologists, intensivists, cardio-vascular surgeons, radiologists, and cardiologists felt that the perforation needs to be addressed along with renal dysfunction, low platelet count (50 × 109/l), and disseminated intravascular coagulation. It was felt that the percutaneous approach is the most appropriate measure considering the less invasive nature of the procedure, short and long term implications, age, and poor general condition of the child.

## Procedure details

**Fig. 1:**
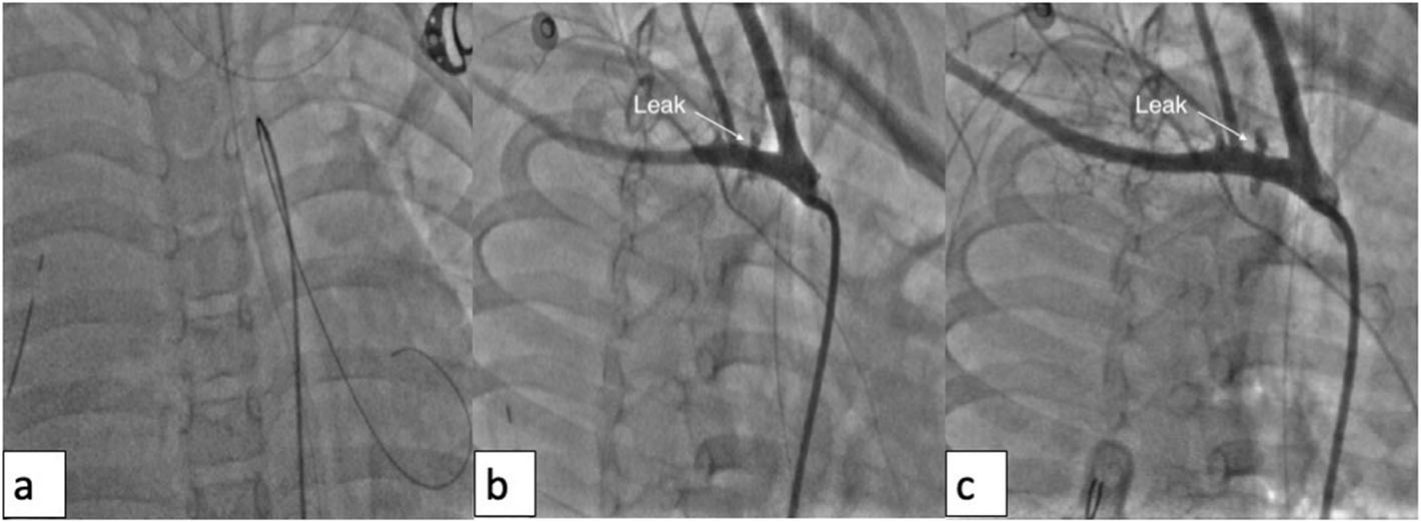
**a** Mediastinal shift to the left with massive right hemothorax. **b** Selective right SCA injection (RAO) showing perforation in the first part, between right CCA, and right vertebral artery. **c** Post ICD revision, selective right SCA injection showing more prominent dye leak s/o significant perforation in the first part, between right CCA, and right vertebral artery

**Fig. 2:**
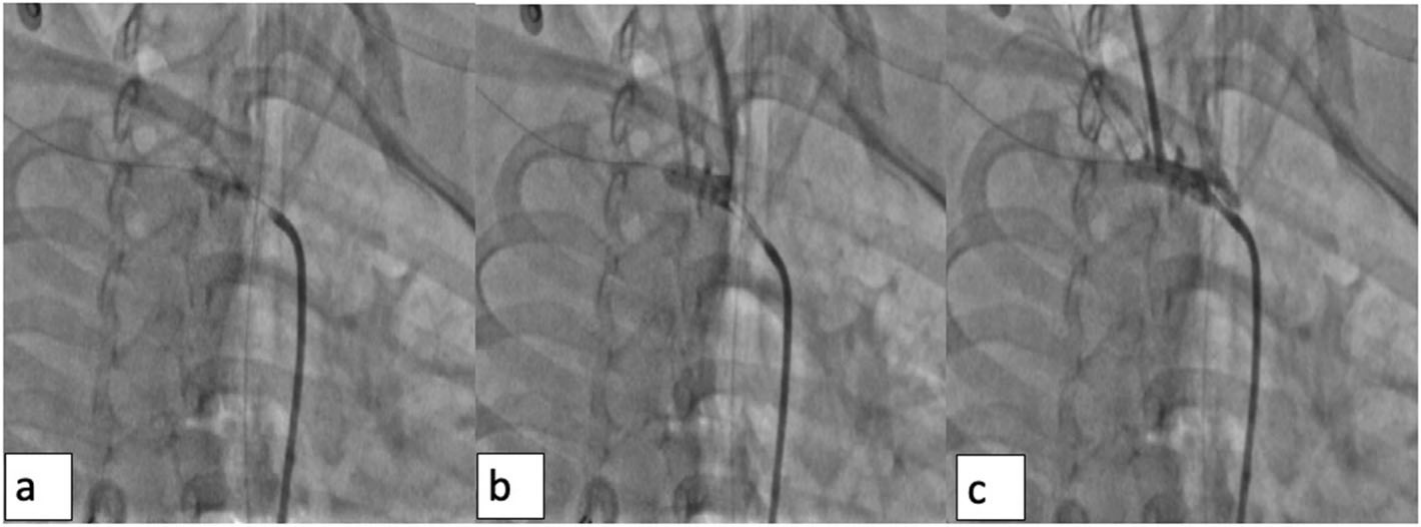
**a** BMW 0.014″ guidewire parked in the right axillary artery. **b** 3.0 × 12, 3.5 × 12, 4.0 × 12 mm SC/NC coronary balloons to occlude the perforation by balloon tamponade effect. **c** Persistent dye leak, despite inflated balloon

**Fig. 3:**
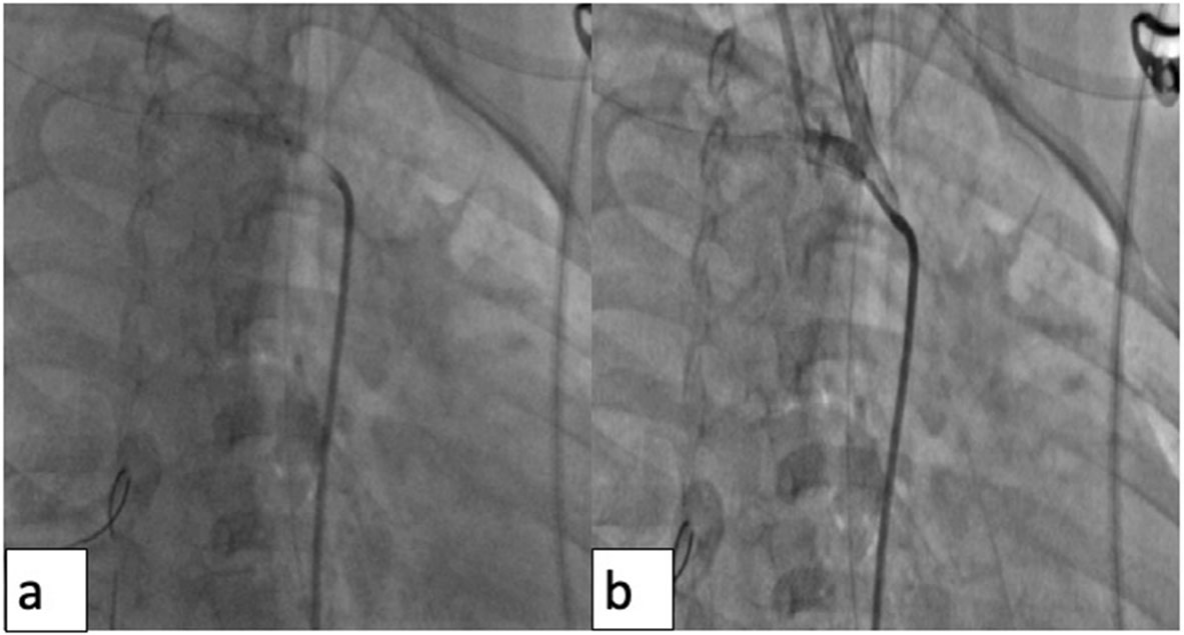
**a** 4 × 16 mm covered stent deployed at 22 atm. **b** Right brachiocephalic injection showing persistent dye leak, although less

**Fig. 4:**
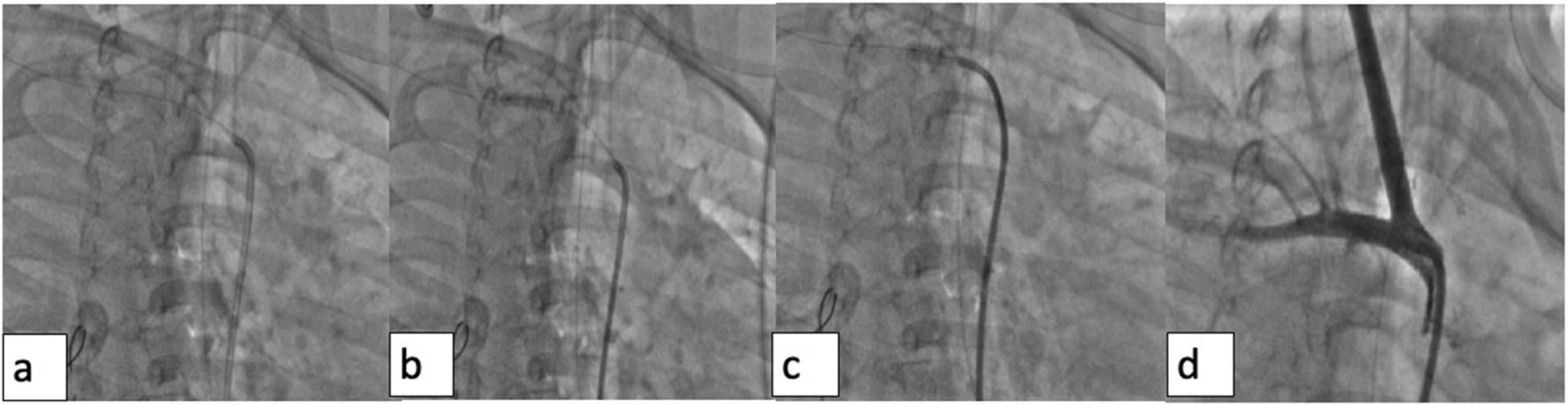
**a** While the stent balloon was being withdrawn, the stent also migrated proximally, just at the origin of the right CCA. **b** Guide catheter pushed, and the stent is moved beyond right CCA, partially inflated 5.0 × 14 mm NC balloon was used to position the stent in between right CCA and vertebral artery. **c** Post-dilatation using 5 × 12 and 5.5 × 12 mm NC balloons to ensure good apposition of the stent with a stable position. **d** Injection showing well apposed stent and no dye leak

Arterial access (left femoral artery, as the right femoral vein was used for hemodialysis catheter, for ease of access left side was used) was gained using 6 Fr sheath. Fluoroscopy of the thorax showed mediastinal shift to the left (Fig. 1a) altering the anatomy of the aortic arch and neck vessels. Selective injection of the right brachiocephalic artery (6 Fr Judkins right catheter, Judkins right was used for selective injections) showed contrast leak from the first part of the right subclavian artery between CCA and VA (Fig. 1b). Although the leak appreciated was minimal, questioning the significance of bleed and intervention in such a small child, we could not rule out the “tamponade effect” by the large hemothorax which could be misleading. Hence, we replaced the existing intercostal draining tube (ICD) with a wide bore one. As the child had hypotension following ICD revision, resuscitation measures were carried out simultaneously with packed cell transfusion. A repeat injection showed a significant contrast leak from the first part of right subclavian artery (Fig. 1c). It was decided to attempt a “balloon tamponade” to tackle this peroration with the hope to avoid stenting in a small child. A 0.014″ BMW wire (Abbott Vascular TM) was parked in the right axillary artery, and sustained inflations were given serially (3.0, 3.5, and 4.0 mm balloons) (Fig. 2a, b). As repeat injection during balloon inflation showed persistent contrast leak (Fig. 2c), it was decided to proceed with a covered stent. The most appropriate size of covered stent available at that time was a 4 × 16 mm GRAFTMASTER (ABBOTT VASCULAR TM) which was deployed at 22 atm (Fig. 3a). We had to face a few more challenges than just hemodynamic support in this case as described below. While the deflated stent balloon was being withdrawn, the stent migrated proximally getting stationed at the origin of right CCA (Fig. 4a) raising concern of cerebral flow compromise. An attempt was made to push the migrated stent distally beyond the ostium of CCA with the help of a partially inflated balloon (Fig. 4b) which sorted out the issue. The partially inflated 5.0 × 14 mm non-compliant (NC) balloon was used to position the stent in between CCA and VA (Fig. 4c). Later, it was post dilated with 5.0 mm and 5.5 mm NC balloon with good apposition (Fig. 4d). Repeat injection showed no contrast leak. The hemodynamic status improved with no requirement of blood products after the procedure. No neurological complications were seen. He was extubated 2 days later. His renal dysfunction and supportive management were continued under expert care. Single antiplatelet was advised after the platelet count improved to more than 100 × 10^9^/L.

## Discussion

Central venous catheter provides multiple venous access in critically ill patients for hemodynamic monitoring, fluid resuscitation, administration of inotropes, nutritional support, and hemodialysis [2]. The complication rate is about 15% including mechanical (arterial puncture, pneumothorax, and hematoma), infectious, and thrombotic complications [1]. Ultrasound guidance reduces the risk of mechanical complication, procedure failure, and shortens the time required for insertion [2]. Arterial injury occurs in around 0.5 to 3.7% of cases in the form of hematoma, hemothorax, pseudoaneurysm, and arteriovenous fistula. The most common arterial injury associated with IJV cannulation is carotid artery puncture. Subclavian artery injury is a rare complication. The typical clinical presentation is a triad of IJV cannulation, hypotension, and X-ray evidence of hemothorax [3, 4]. Management guidelines in cases of arterial injury are not very clear. Open surgical repair involves extensive surgical dissection in critically ill patients. Percutaneous management offers a less invasive, less time consuming alternative in critically ill patients in emergencies. Literature search showed only a few case reports of percutaneous management of arterial injury following IJV cannulation. In one of the cases, the misplaced catheter intended to be put in IJV was detected on chest X-ray. When the catheter was removed after 2 days, there was a hemodynamic compromise that was managed with stent graft [4]. In another case, a vascular plug was used to close the arterial injury that occurred during subclavian venous puncture [5]. Comparison of percutaneous approach with open surgical approach found to be non-inferior in terms of success and better in safety. In a retrospective case series where endovascular approach using stent graft was compared with surgical approach for subclavian artery injuries (three of them caused by subclavian artery catheterization attempts), it was shown that blood loss (70 ± 12.2 mL vs 220 ± 56.1 mL; *P* < 0.01) and procedural time (132 ± 15 min vs 193 ± 15 min; *P* = 0.04) was lesser in endovascular group [6]. There was no difference in patency rates between the two groups [6]. Endovascular treatment obviates the need for surgical dissection preventing injury to the adjacent structures like vagus nerve, recurrent laryngeal nerve, phrenic nerve, and innominate vein [6]. Careful selection of the patient is necessary as lesions that are focal and are at a good distance from the vertebral artery are amenable for endovascular therapy. In our case, we had a sick child with HUS, thrombocytopenia, and acute renal failure requiring renal replacement therapy had hemothorax following IJV catheter placement leading to hemodynamic instability which was managed with multiple blood products transfusions. In such cases, open repair is difficult due to critically ill status and severe thrombocytopenia. We went ahead with the endovascular approach as it is less invasive and less time consuming. We encountered the problems of undersized stent and its poor apposition to the vessel wall leading to stent migration which was successfully managed. Using stent in a growing child has its implications like in-stent restenosis; vessel growth to stent size remains a challenge in the future, though development of collaterals would ensure adequate limb growth and functioning.

## Conclusion

Arterial injury although rare is a potentially life-threatening complication of IJV cannulation which warrants immediate attention and correction. Ultrasound guidance during central venous catheter placement helps in avoiding mechanical complications. The endovascular technique offers a less invasive approach with less morbidity in critically ill and emergencies.

## Supplementary information

**Additional file 1: Video 1.** Selective angiogram of right subclavian artery showing contrast leak from the first part of subclavian artery between right common carotid artery and vertebral artery.

**Additional file 2: Video 2.** Fluoroscopy showing covered stent being deployed at the site of leak.

**Additional file 3: Video 3.** Final angiogram showing sealed perforation with no compromise of branch vessels.

## Data Availability

The datasets used and/or analyzed during the current study are available from the corresponding author on reasonable request.

## Abbreviations

BMW: Balance middle weight
CCA: Common carotid artery
CT: Computed tomography
ICD: Intercostal drainage tube
IJV: Internal jugular vein
NC: Non-compliant
VA: Vertebral artery
